# YY1 Acts as a Transcriptional Activator of *Hoxa5* Gene Expression in Mouse Organogenesis

**DOI:** 10.1371/journal.pone.0093989

**Published:** 2014-04-04

**Authors:** Félix-Antoine Bérubé-Simard, Christelle Prudhomme, Lucie Jeannotte

**Affiliations:** 1 Department of Molecular Biology, Medical Biochemistry and Pathology, Université Laval, Québec, Canada; 2 Centre de recherche sur le cancer de l′Université Laval, Québec, Canada; 3 Centre de recherche du Centre Hospitalier Universitaire de Québec, L'Hôtel-Dieu de Québec, Québec, Canada; Cincinnati Children's Hospital Medical Center, United States of America

## Abstract

The *Hox* gene family encodes homeodomain-containing transcriptional regulators that confer positional information to axial and paraxial tissues in the developing embryo. The dynamic *Hox* gene expression pattern requires mechanisms that differentially control *Hox* transcription in a precise spatio-temporal fashion. This implies an integrated regulation of neighbouring *Hox* genes achieved through the sharing and the selective use of defined enhancer sequences. The *Hoxa5* gene plays a crucial role in lung and gut organogenesis. To position *Hoxa5* in the regulatory hierarchy that drives organ morphogenesis, we searched for *cis*-acting regulatory sequences and associated *trans*-acting factors required for *Hoxa5* expression in the developing lung and gut. Using mouse transgenesis, we identified two DNA regions included in a 1.5-kb *Xba*I-*Xba*I fragment located in the *Hoxa4-Hoxa5* intergenic domain and known to control *Hoxa4* organ expression. The multifunctional YY1 transcription factor binds the two regulatory sequences *in vitro* and *in vivo*. Moreover, the mesenchymal deletion of the *Yy1* gene function in mice results in a *Hoxa5*-like lung phenotype with decreased *Hoxa5* and *Hoxa4* gene expression. Thus, YY1 acts as a positive regulator of *Hoxa5* expression in the developing lung and gut. Our data also support a role for YY1 in the coordinated expression of *Hox* genes for correct organogenesis.

## Introduction

*Hox* genes encode evolutionarily conserved transcription factors that control the formation of body segment-specific structures by regulating the transcription of downstream effectors that, in turn, direct the morphogenetic events leading to the complex body forms along the embryonic axes in metazoan [1]–[2]. Consequently, mutations in *Hox* genes alter segmental identity and cause morphological defects. In mammals, 39 *Hox* genes are distributed over four clusters, each containing 9 to 11 genes closely packed in less than 150-kb of sequences (*HoxA* to *D*). Their spatio-temporal expression profile during embryogenesis reflects their arrangement in the clusters: the 3′ most genes are expressed earlier and their expression domain reaches a more anterior limit than those occupying 5′ positions. As a result, members of the *Hox* complexes are expressed in nested and overlapping domains along the developing body suggesting that specific combinations of HOX proteins provide a unique address to defined regions [3]. Based on sequence homology and location within clusters, *Hox* genes are also classified into 13 paralog groups.

The *Hox* clustered organization is fundamental for the precise regulation and the function of each gene and hence for the correct formation of the embryo. Analysis of *Hox* mutant mice endorses the collinear relationship between the position of individual genes within *Hox* clusters and the structural defects observed along the anterior-posterior (A-P) axis [4]. For example, the mutation of the *Hoxa5* gene, located in the middle of the *HoxA* multigenic complex, affects axial specification at the cervico-thoracic level [5]. A high percentage of *Hoxa5^-/-^* pups die at birth from impaired respiratory tract development [6]. Moreover, the loss of *Hoxa5* function results in panoply of phenotypes indicative of the broad range of *Hoxa5* actions throughout life [7]–[11]. Most defects in *Hoxa5^-/-^* mutants are confined to the cervico-thoracic region corresponding to the *Hoxa5* rostral-most expression domain, where the major *Hoxa5* transcript of 1.8-kb encoding the HOXA5 270-amino-acid protein, is specifically expressed [12]. Thus, *Hoxa5* appears as a critical determinant in the specification and the development of a subset of structures at the cervico-thoracic level.

While the developmental role of *Hox* genes is well established, the regulation of *Hox* gene expression in the embryo remains incompletely understood. A complex array of different modes of regulation governs the precise *Hox* expression [13]–[14]. Regulation primarily occurs at the transcriptional level via the combinatorial interplay of several signaling pathways and transcriptional factors that interact with positive and negative *cis*-acting sequences to differentially control *Hox* expression in a spatio-temporal and tissue-specific fashion. The proximity of *Hox* genes in clusters implies the integrated regulation of adjacent *Hox* promoters through the sharing, the competition and/or the selective use of defined enhancers [15]. In parallel, global regulatory elements located outside the *Hox* clusters and able of long-distance action coordinate the expression of several genes along the *Hox* complexes [14]. Large-scale chromatin remodeling events also participate to the regulation of the collinear expression of *Hox* genes [16].

Transcriptional regulators of *Hox* gene expression have been identified [17]. They include developmentally regulated factors like the CDX homeodomain-containing proteins that integrate retinoic acid (RA), FGF and Wnt signaling for the setting of the correct expression domain of *Hox* genes [18]–[21]. *Hox* genes are also directly responsive to RA, which activates retinoic acid receptors that then interact with retinoic acid response elements (RARE) identified near *Hox* genes mainly from paralog groups 1 to 5 [22]–[23]. *Hox* expression is under the control of HOX proteins themselves involved in auto- and cross-regulation [24]–[25]. Finally, ubiquitously expressed transcription factors such as the multifunctional Yin Yang 1 (YY1) protein can modulate *Hox* expression in specific contexts [26]–[29].

A complex organization of overlapping transcriptional units encompassing the *Hoxa5* locus exists, which results from alternative splicing and the use of three promoters, one proximal producing the 1.8-kb transcript and two distal ones giving rise to long noncoding RNAs [12]. Using a transgenic approach, we have identified regulatory elements directing the developmental expression of the *Hoxa5* proximal promoter. An 11.1-kb genomic fragment can recapitulate the temporal expression and substantially reconstitute the spatial pattern of the 1.8-kb *Hoxa5* transcript in mouse embryos. It includes DNA control sequences, such as the 604-bp brachial spinal cord (BSC) enhancer and a 650-bp temporal control region, both contained in the *Hoxa5* 5′ flanking sequences [30]–[31]. A 2.1-kb mesodermal (MES) enhancer important for *Hoxa5* paraxial and lateral plate mesoderm expression in the cervico-upper thoracic region of the A-P axis is positioned 3′ of the *Hoxa5* gene. CDX proteins bind this sequence acting as potential regulators for the regionalization of *Hoxa5* gene expression along the axis [32]. A 1.5-kb DNA region that targets *Hoxa5* lung and gut developmental expression was also identified in the *Hoxa4*-*Hoxa5* intergenic sequences [33].

Several *Hox* genes, mainly from paralog groups 1 to 8, are expressed along the respiratory tract [34]. However except for *Hoxa5*, the lack of overt lung phenotype in single *Hox* mutants indicates that *Hoxa5* plays a predominant function in lung ontogeny [35]. The prevalent role of *Hoxa5* in organ development prompted us to further characterize *Hoxa5* lung and gut regulatory sequences. Here, we present evidence that *Hoxa5* lung and gut expression is under the control of several DNA elements. Some are shared with the flanking *Hoxa4* gene and they bind the transcription factor YY1, which acts as a positive regulator of *Hoxa5* gene expression in the developing lung and gut.

## Materials and Methods

### Ethics statement

All animal experimentations were performed according to the guidelines of the Canadian Council on Animal Care and they were specifically approved by the institutional animal care committee (Comité de protection des animaux du Centre de recherche du Centre Hospitalier Universitaire de Québec (CPAC); Permit Number: 2012013-2).

### Design of *Hoxa5*/*lacZ* transgenes

All *Hoxa5*/*lacZ* constructs contain the bacterial *lacZ* gene inserted into the *Sac*I site of the first exon of the *Hoxa5* gene, which allows translation of the *lacZ* ORF from the HOXA5 AUG [31]. Construct 1 (also named pLJ272) was obtained by adding a 5.20-kb *EcoR*I-*Apa*I DNA fragment at the 3′ end of construct 2 from ref. [33]. Construct 2 (pLJ123) was previously described (construct 6 in ref. [31]). It was used as the backbone for most *Hoxa5*/*lacZ* constructs in the present study. Constructs 3, 4, 8–12, 14–15 and 17–21 were obtained by ligation at the 3′ end of pLJ123 of genomic fragments from the *Hoxa4*-*Hoxa5* intergenic region. Construct 5 was produced by cloning the 1.5-kb *Xba*I-*Xba*I fragment upstream an *Xho*I-*Hind*III *Hoxa5* genomic fragment carrying a *Hoxa5* minimal promoter that cannot direct β-galactosidase expression by itself (pLJ143 in ref. [32]). Enhancer activity was tested in constructs 6, 7, 13, 16 and 22 by cloning *Hoxa5* regulatory regions in front of the *hsp68lacZpA* fragment, which contains an *hsp68* minimal promoter that cannot direct *lacZ* developmental tissue-specific expression by itself [31], [36]. For constructs 17 to 22, nucleotide substitutions in RARE and YY1 binding sites were produced by an overlapping PCR strategy using synthetic oligonucleotide primers carrying appropriate base changes (Table 1A). Introduced mutations were confirmed by sequencing and disruption of the binding sites was validated with the TFSEARCH (http://molsun1.cbrc.aist.go.jp/research/db/TFSEARCH.html) and TESS (http://www.cbil.upenn.edu/cgi-bin/tess/tess) softwares.

**Table 1:** Oligonucleotide sequences.

Fragment		Sequence (5′-3′)	Amplicon (bp)
**A) Mutagenesis**			
259-bp *Xba*I-*BssH*II (mRARE)		F-GCGCAAAGTCCAAGGCCGTGGTGCACTT CTAGTCGGCGCGTTCTAA	46
259-bp *Xba*I-*BssH*II (mYY1)		F-GACCCTGTCGGCCGCCCAGGGC	22
163-bp *Nco*I-*Sac*I (mYY1)		F-CAGTACCCTGTCACCCTGTCGG	22
**B) EMSA on the 259-bp ** ***Xba*** **I-** ***Bss*** **HII DNA fragment (C3)**			
Oligo RARE	F-GCGCAAAGTCCAAGGCCGAGGTGAACTT CAGGTCAGCGCGTTCTAA		46
Oligo mRARE	F-GCGCAAAGTCCAAGGCCGTGGTGCACTT CTAGTCGGCGCGTTCTAA		46
Fragment A	F-CTAGAGAAGTGTTTGAGATAG R-CATTATGGGATGTATTGACTG		85
Fragment B	F-CAGTCAATACATCCCATAATG R-CAGTGTCTTTTAGAGAGCTGG		104
Fragment C	F-CCAGCTCTCTAAAAGACACTG R-CCCTCACCAGCCGACATTTTC		110
Oligo C1	F-CCCAGCTCTCTAAAAGACACTGTATAGA CCTTTTAGAAGCG		41
Oligo C2	F-AAGCGCAAAGTCCAAGGCCGAGGTGAACT TCAGGTCAGCG		40
Oligo C3	F-CAGCGCGTCTAACAAATATGAAAATGTC GGCTGGTGAGGGCGCG		44
Oligo C3(m1)	F-GCTATATTATAACAAATATGAAAATGTC GGCTGGTGAGGGCGCG		44
Oligo C3(m2)	F-CAGCGCGTCGCCACCCGCTGAAAATGTC GGCTGGTGAGGGCGCG		44
Oligo C3(m3)	F-CAGCGCGTCTAACAAATAGTCCCCGTGC GGCTGGTGAGGGCGCG		44
Oligo C3(m4)	F-CAGCGCGTCTAACAAATATGAAAATGTA TTAGTTGT AGGGCGCG		44
Oligo C3(m5)	F-CAGCGCGTCTAACAAATATGAAAATGTC GGCTGGTGCTCTATAC		44
Oligo-18(C3)	F-TGAAAATGTCGGCTGGTG		18
Oligo C3(mYY1)	F- TCAGCGCGTCTAACAAATATGACCCTGTC GGCCGCCCAGGGCGCG		45
Oligo C3(m1YY1)	F-TCAGCGCGTCTAACAAATATGACCCTGTC GGCTGGTGAGGGCGCG		45
Oligo C3(m2YY1)	F-TCAGCGCGTCTAACAAATATGAAAATGTC GGCCGCCCAGGGCGCG		45
Consensus YY1	F-GGGGATCAGGGTCTCCATTTTGAAGCGG GATCTCCC		36
**C) EMSA on the 433-bp ** ***Mef*** **I-** ***Sac*** **I DNA fragment (G3)**			
Oligo G1	F-CATGGGATTTTTGCTATGGCTTGCTTGCA AAGGGAGGCTGTGGAA		45
Oligo G2	F-TGGAAGGTTCAGGAAAGGTACTGAGATT GTTTATTACAGCCATAA		45
Oligo G3	F-CATAAATCTTGCAGTAAAATGTCAAAAT GTCGGTGTGTGAGATAA		45
Oligo G4	F-GATAACACTTGGTGGTCCTGGCTCCGTTT GTGTTTATGATAGGAGCT		47
Oligo G3(m1)	F-GCGCCCCAGTGCAGTAAAATGTCAAAAT GTCGGTGTGTGAGATAA		45
Oligo G3(m2)	F-CATAAATCTGTACTGCCGATGTCAAAAT GTCGGTGTGTGAGATAA		45
Oligo G3(m3)	F-CATAAATCTTGCAGTAAAGGTAAGTCCT GTCGGTGTGTGAGATAA		45
Oligo G3(m4)	F- CATAAATCTTGCAGTAAAATGTCAAAACTGA TTGTGGTGAGATAA		45
Oligo G3(m5)	F-CATAAATCTTGCAGTAAAATGTCAAAAT GTCGGTGTTGTGTCGCC		45
Oligo-18(G3)	F-ATGTCAAAATGTCGGTGT		18
Oligo G3(mYY1)	F-CATAAATCTTGCAGTACCCTGTCACCCTG TCGGTGTGTGAGATAA		45
Oligo G3 (m1YY1)	F-CATAAATCTTGCAGTACCCTGTCAAAATG TCGGTGTGTGAGATAA		45
Oligo G3 (m2YY1)	F-CATAAATCTTGCAGTAAAATGTCACCCTG TCGGTGTGTGAGATAA		45
**D) ChIP**			
C3	F-ACTTCAGGTCAGCGCGTCTAACAA R-CACTTGAATGCAACCCTGTCCCAA		161
G3	F-TTGCTATGGCTTGCTTGCAAAGGG R-CCTGTAACACGTCTTTGAGCTCCT		173
15 kb 3′	F-TGAAGTGTGAAGGTGCAGCAAACG R-AAAGGTGAGACCTTGCAGAGCAGA		140
*Sfrs10*	F-TTTCTCCGCTTCACCCTTGGA R-AACGGTATCTTCTTTCGCCGTTGGA		175
*Rcor3*	F-GTCTCAGCTGAAGGAATTTGGCCT R-GCCATCCTCATAGCTCCTGTCAAA		157
**E) qRT-PCR**			
*Hoxa4*	F-CGCCGTCAACTCCAGTTAT R-AGTGGAATTCCTTCTCCAGTTC		95
*Hoxa5*	F-CCCAGATCTACCCTGGATG R-GGCATGAGCTATTTCGATCCT		173
*Rpl19*	F-GATCATCCGCAAGCCTGTGA R-GCATCCGAGCATTGGCAGTA		122
*Scgb1a1*	F-AAGCCTCCAACCTCTACCATG R-ATGTCCGAAGAAGCTGAGCTG		85
*T1α*	F-TGGCAAGGCACCTCTGGTA R-GGTGGACAGTTCCTCTAAGGGA		70
*Yy1*	F-CATGTGGTCCTCGGATGAAA R-GGGAGTTTCTTGCCTGTCATA		117

### Production of transgenic mice

The microinjected *Hoxa5/lacZ* and *hsp68lacZpA* fragments were obtained by a *Sal*I+*Not*I digestion and an *Xho*I+*Xba*I digestion, respectively, to eliminate plasmid sequences. Transgene microinjection into fertilized eggs from C57BL/6 X CBA F1 hybrid intercrosses were done according to standard procedures [37]. Transgenic founder embryos were recovered from foster mothers, genotyped by Southern analysis of yolk sac DNA using a *lacZ* specific probe to assess the integrity of the microinjected construct, and analyzed for *lacZ* expression by β-galactosidase staining. Permanent mouse lines were also obtained for construct 1 and genotyped with tail DNA [31].

### Mice, genotyping, tissue collection, histology, immunohistochemistry (IHC) and β-galactosidase staining

To assess the effect of YY1 on *Hoxa5* expression, mutant mouse lines for the *Yy1* ^flox^ and *Yy1* ^Floxneo^ alleles were used (provided by Y. Shi) [38]. For the mesenchymal ablation of *Yy1*, we used *Dermo1* ^Cre^ mice provided by D. Ornitz [39]. *Dermo1* ^Cre^ mice express the Cre recombinase protein from the endogenous *Dermo1* (*Twist2*) locus. *Yy1* ^flox/flox^ *Dermo1* ^+/Cre^ mice were generated by matings between *Yy1* ^flox/+^ *Dermo1* ^+/Cre^ and *Yy1* ^flox/flox^ mice.

Age of the embryos was estimated by considering the morning of the day of the vaginal plug as embryonic (E) day 0.5. Experimental specimens were genotyped by Southern blot and PCR analyses. Organs were collected from E13.5, E15.5 and E18.5 wild-type (wt), *Hoxa5/lacZ* transgenic and mutant embryos. For RNA extraction, tissues were snap-frozen in N_2_. For histology and immunostaining, tissues were fixed in 4% ice-cold paraformaldehyde (PFA), paraffin-embedded and sectioned at 4 μm [40]. Organ sections were stained according to standard hematoxylin and eosin procedure for morphology analysis. Immunohistochemistry was performed as described [11]. The primary antibodies used were a goat antibody against CC10 (1/500; gift for Dr. G. Singh), a syrian hamster antibody against podoplanin (T1α; 1/75; DSHB), and a rabbit antibody against YY1 (1/500; Santa Cruz Biotechnology). The biotinylated secondary antibodies used were a goat anti-rabbit antibody (1/300; Vector Laboratories), a swine anti-goat antibody (1/300; Cedarlane), and a goat anti-syrian hamster antibody (1/300; Jackson Immuno Research).

For whole-mount detection of β-galactosidase activity, E8.5 to E13.5 entire embryos and dissected organs from E13.5 embryos were fixed in ice-cold 0.25% glutaraldehyde in PBS for 15 min to one hr according to the age and/or size of the sample. Detection of β-galactosidase activity was performed as described [31]. In parallel, organs from E13.5 embryos were fixed one hr in ice-cold 4% PFA, 0.2% glutaraldehyde in PBS, put overnight in a 30% sucrose solution in 0.1M phosphate buffer pH8.0, and embedded in tissue freezing medium (Triangle Biomedical Sciences, Inc.). Ten μm cryosections were processed for *lacZ* expression and counterstained with nuclear Fast Red [41].

### Electrophoretic mobility shift assay (EMSA) and supershift assay

Whole cell extracts (WCE) were prepared from organs (lung, stomach and intestine) of E13.5 wt mouse embryos as described [19], [32]. The expression vector pCDNAI-YY1 (provided by G. Blanck) was used with the TnT7 Quick coupled transcription-translation system (Promega) to produce *in vitro* YY1 protein that was analyzed on sodium dodecyl sulfate-polyacrylamide gel electrophoresis. Both WCE and YY1 protein were tested in EMSA.

The 259-bp *Xba*I-*Bss*HII and the 433-bp *Mef*I-*Sac*I DNA fragments were radiolabelled from digested plasmids with the Klenow fragment and purified on a G-50 Sephadex column. These DNA probes were separated on non-denaturating 6% polyacrylamide (29:1) gels, from which bands of interest were cut and electroeluated. Binding reactions containing 0.5 to 2 ng of probe (2,000 cpm), 5 μg of WCE, 1 μg of poly(dI-dC), and 10 μg of BSA were prepared in 5 mM Hepes pH 7.9, 10% glycerol, 25 mM KCl, 0.05 mM EDTA and 0.125 mM PMSF. Samples were equilibrated for 10 min at room temperature and separated by electrophoresis through a 6% polyacrylamide (29:1) gel prepared in 0.25X Tris-borate-EDTA buffer. Binding specificity was assessed by addition of a 100-fold excess of unlabelled probe or competitor prior to addition of the radiolabelled probe.

Oligonucleotides were also used to generate radiolabelled probes and cold competitors (Table 1B–C). Radiolabelling was achieved with the T4 polynucleotide kinase (NEB). Binding reactions using these probes contained 0.5 to 2 ng (50,000 cpm), 2 μl of YY1 protein produced *in vitro* or 5 μg of WCE, 1 μg of poly(dI-dC), and 10 μg of BSA (NEB) prepared in 15 mM Hepes pH7.9, 50 mM Nacl, 80 μM ZnCl_2_, 800 μM dithiothreitol, 0.5% NP-40, 2 mM MgCl_2_, and 3% Ficoll. Samples were equilibrated for 5 min at room temperature. In supershift assays, 2 μg of YY1 antibody was added for an additional 15 min. Reaction products were separated by electrophoresis through a 6% polyacrylamide (29:1) gel prepared in 0.25X Tris-borate-EDTA and 2.5% glycerol buffer. Binding specificity was validated using only rabbit reticulocyte extract as negative control. The supershift specificity was assessed using as negative control a CDX2 antibody (provided by D. Lohnes).

### Chromatin immunoprecipitation (ChIP) assays

Lung and stomach from E13.5 wt embryos were collected separately and mechanically disrupted to produce a single cell suspension prior cross-linking with 1% formaldehyde in PBS for 15 min at room temperature. Cross-linking was stopped by adding glycine to a final concentration of 0.125 M. Extracts were then disrupted into crude lysates in 2 ml of swelling buffer (5 mM Pipes pH8.0, 85 mM KCl, 1% NP-40 and protease inhibitors), equilibrated 20 min on ice, and centrifuged for 5 min at 3000 rpm at 4°C. The pellets were eluted in 1 ml of nuclear lysis buffer (50 mM Tris-HCl pH 8.0, 10 mM EDTA, 1% SDS) and protease inhibitors (Complete Mini-EDTA-free; Roche Diagnostics) and submitted to two successive rounds of sonication using a Bioruptor (Diagenode) for a total of 10 cycles of a 30-sec pulse interrupted by a 30-sec pause at the highest setting in order to obtain an average DNA size of 300-600-bp. One hundred μg of fragmented chromatin was incubated overnight at 4°C with Dynabeads linked to protein G (Invitrogen) and 2 μg of rabbit anti-YY1 antibody, rabbit anti-H3 antibody (ab1791; Abcam), or control rabbit IgG (sc2027; Santa Cruz Biotechnology). Immunocomplexes were washed three times respectively with 0.5 ml of low salt immune complex wash buffer (0.1% SDS, 1% Triton X-100, 2 mM EDTA, 20 mM Tris-HCl pH 8.0, 150 mM NaCl), high salt immune complex wash buffer (0.1% SDS, 1% Triton X-100, 2 mM EDTA, 20 mM Tris-HCl pH 8.0, 500 mM NaCl) and LiCl immune complex wash buffer (0.25 M LiCl, 1% IGEPAL-CA630, 1% deoxycholic acid (sodium salt), 1 mM EDTA, 10 mM Tris-HCl pH 8.0). A final wash in 0.5 ml of TE buffer (10 mM Tris-HCl pH 8.0, 1 mM EDTA) was followed by centrifugation at 3000 rpm at 4°C for 2 min. Protein-DNA complexes were eluted by adding 100 μl of elution buffer (1% SDS, 50 mMTris pH 8.0, 10 mM EDTA) and then equilibrated at 65°C for 15 min. Cross-links were reversed by adding 200 mM NaCl and equilibrated at 65°C overnight. DNA fragments were purified using a QIAquick gel extraction kit (Qiagen) after a two hr treatment with RNase followed by a two hr treatment with proteinase K. qPCR analyses were performed with specific primers listed in Table 1D. The values for the samples immunoprecipitated by anti-YY1, anti-histone H3 and control IgG were recorded as the percentage relative to input. ChIP results were confirmed by three independent experiments and qPCR was performed in triplicate for each sample. The ChIP efficiency was calculated by dividing the amount of PCR product obtained with the immunoprecipitated DNA by the amount obtained with the input DNA, as described in ref. [42].

### Quantitative RT-PCR (qRT-PCR) experiments

Total RNA was isolated from lungs of E18.5 embryos. RNA extraction, cDNA synthesis and qRT-PCR were performed as described [41]. Samples were analyzed in triplicate. The *Rpl19* gene was used as control. Eight specimens were used for each genotype tested. Primer sequences are listed in Table 1E.

### Statistical analyses

Student's *t* test was performed for gene expression and ChIP studies. A significance level inferior to 5% (*p*<0.05) was considered statistically significant.

## Results

### A 14.5-kb genomic fragment recapitulates the spatio-temporal expression from the *Hoxa5* proximal promoter

**Figure 1:**
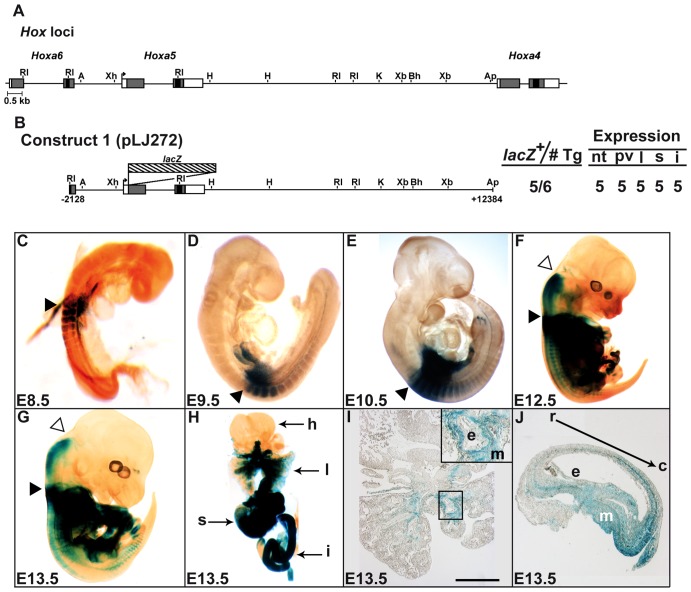
β-galactosidase expression profile of construct 1 during embryogenesis. (A) Partial restriction map of the *Hoxa4-Hoxa6* genomic region. Black boxes represent homeoboxes, shaded boxes correspond to translated regions, and white boxes indicate transcribed regions. The arrow shows TSS from the *Hoxa5* proximal promoter. (B) Diagram of the *Hoxa5/lacZ* construct 1 (also named pLJ272) and summary of transgenic expression analysis at E13.5. The first column presents the number of *lacZ*-expressing F0 embryos out of the total number of F0 transgenic embryos generated. The number of positively stained embryos for each structure listed is indicated. A, *Acc*I; Ap, *Apa*I; Bh, *Bss*HII; RI, *Eco*RI; H, *Hind*III; K, *Kpn*I; Xb, *Xba*I; Xh, *Xho*I. (C–G) Representative transgenic embryos stained for β-galactosidase activity showed the expression pattern of construct 1 during development. Black arrowheads indicate the anterior limit of transgene expression in paraxial mesoderm at somite 5 and prevertebra 3. Open arrowheads point the anterior limit of transgene expression in the neural tube. (H) Whole-mount organs from E13.5 transgenic embryos showed β-galactosidase activity in lung, stomach and intestine. (I–J) Histological sections of lung and stomach, respectively, from E13.5 transgenic embryos stained for β-galactosidase activity. The inset in I shows that expression was restricted to mesenchyme. Black arrow in J shows the gradient of expression of the transgene in the developing stomach. c, caudal; e, epithelium; h, heart; i, intestine; l, lung; m, mesenchyme; nt, neural tube; pv, prevertebrae; r, rostral; s, stomach. Scale bar: 100 μm.

We have previously shown that the 11.1-kb *Hoxa5* genomic region located between positions -3767-bp and +7188-bp (relative to *Hoxa5* transcription start site (TSS)) reproduced the temporal expression and substantially reconstituted the spatial profile of *Hoxa5* gene. However, it did not recapitulate the correct expression in the central nervous system (CNS) and in developing organs, indicating that additional sequences were required [31]. A 1.5-kb *Xba*I-*Xba*I DNA fragment located in *Hoxa4*-*Hoxa5* intergenic sequences at ∼3.0-kb upstream the *Hoxa4* gene (positions +9351-bp to +10816-bp) was able to target *Hoxa5* expression in lung and stomach [33]. To assess if a DNA region encompassing the *Hoxa5* regulatory sequences identified can recapitulate *Hoxa5* expression from the proximal promoter, a 14.5-kb *Hoxa5/lacZ* transgene (positions -2128-bp to +12384-bp) was tested (Construct 1; Fig. 1A–B). As reported for the 11.1-kb *Hoxa5/lacZ* transgene, the onset of expression of construct 1 was ∼E8.0-8.25, corresponding to that of *Hoxa5* endogenous expression (not shown) [31], [43]. At E8.5, staining was mainly detected in the foregut region and in somites 5 to 8, consistent with the *Hoxa5* expression domain in the mesoderm along the A-P axis at this age (Fig. 1C) [31]. At E9.5-10.5, transgene expression extended more caudally along the axis and appeared in limb buds (Fig. 1D–E). At E12.5-13.5, staining was detected along the neural tube with an anterior boundary in the posterior hindbrain corresponding to the limit of *Hoxa5* endogenous expression in CNS. No staining was seen in the most caudal part of the embryo, which expressed the larger *Hoxa5* transcripts from the distal promoters (Fig. 1F–G) [12]. Construct 1 directed transgene expression in lung, stomach and intestine, and the staining was mainly restricted to the mesenchymal layer as *Hoxa5* endogenous expression (Fig. 1H–J). The *Hoxa5* rostro-caudal gradient present in the developing stomach was reproduced with construct 1 (Fig. 1J) [8]. *lacZ* staining was detected in the intestine, being stronger in the proximal part of midgut and vanishing towards the hindgut (not shown). Thus, the 14.5-kb *Hoxa5/lacZ* transgene contains regulatory sequences that largely reproduce the *Hoxa5* spatio-temporal expression driven by the *Hoxa5* proximal promoter.

### *Hoxa5* organ-specific expression involves several regulatory sequences

**Figure 2:**
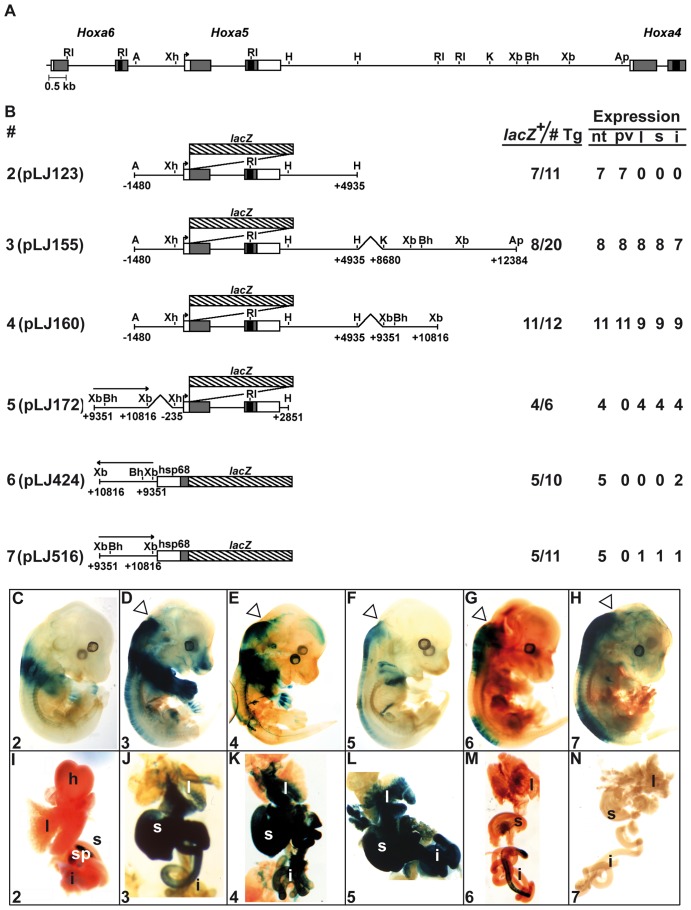
Characterization of the *Hoxa4-Hoxa5* intergenic region in E13.5 F0 transgenic mouse embryos. (A) Schematic representation of the 1.5-kb *Xba*I-*Xba*I DNA fragment in the *Hoxa4-Hoxa6* genomic region. (B) Diagram of the *Hoxa5/lacZ* constructs used to generate E13.5 F0 transgenic embryos and summary of transgenic expression analyses. A, *Acc*I; Ap, *Apa*I; Bh, *Bss*HII; Bm, *Bsm*I; RI, *EcoR*I; H, *Hind*III; K, *Kpn*I; Mf, *Mfe*I; Nc, *Nco*I; Sc, *Sac*I; Xb, *Xba*I; Xh, *Xho*I. (C–H) Carcass of representative E13.5 transgenic embryos and the associated organs (I–N) stained for β-galactosidase activity showed the effects of the different deletions on the expression pattern. Open arrowheads point the anterior limit of transgene expression in the neural tube. The number in the lower left corner of each panel corresponds to the transgene. h, heart; i, intestine; l, lung; nt, neural tube; pv, prevertebrae; s, stomach; sp, spleen.

To delineate the DNA regions involved in *Hoxa5* organ-specific expression, we analyzed shorter versions of the *Hoxa4*-*Hoxa5* intergenic region in E13.5 transgenic embryos (Fig. 2A–B). Construct 2 (pLJ123) was used as control. It contains the BSC and MES enhancer sequences. It also drives expression in forelimbs and spleen but not in lung and gut (Fig. 2C, I) [31], [33]. Insertion of the 3.7-kb *Kpn*I-*Apa*I genomic fragment downstream of pLJ123 reproduced the spatial profile of construct 1 at the same age (construct 3; Fig. 2D, J). A similar result was obtained when the 1.5-kb *Xba*I-*Xba*I DNA fragment was inserted at the 3′ end of pLJ123 indicating that the 1.5-kb sequence contained the regulatory information necessary for proper *Hoxa5* expression in organs (construct 4; Fig. 2E, K). Histological analyses of stained organs from construct 4-positive embryos revealed that expression was restricted to mesenchyme as seen with construct 1 (not shown). To assess if the 1.5-kb DNA region possesses enhancer properties, we put it in front of a *Hoxa5/lacZ* minimal plasmid (construct 5) and of a heterologous *hsp68*/*lacZ* plasmid (constructs 6, 7), both plasmids being unable to direct transgene expression on their own [30], [32], [36]. Construct 5 targeted a strong transgene expression in organs and CNS reinforcing the notion that tissue-specific sequences for these structures were present in the 1.5-kb sequence (Fig. 2F, L). In contrast, when tested in the *hsp68*/*lacZ* context, faint or no organ expression was observed while CNS expression was reproduced (Fig. 2G–H, M–N). Thus, the 1.5-kb *Xba*I-*Xba*I region encloses several *Hoxa5* regulatory elements including a CNS-specific enhancer that directs the appropriate limit of expression in the posterior hindbrain regardless of the promoter used, and an organ-specific sequence that requires a *Hoxa5* minimal environment for effectiveness.

**Figure 3:**
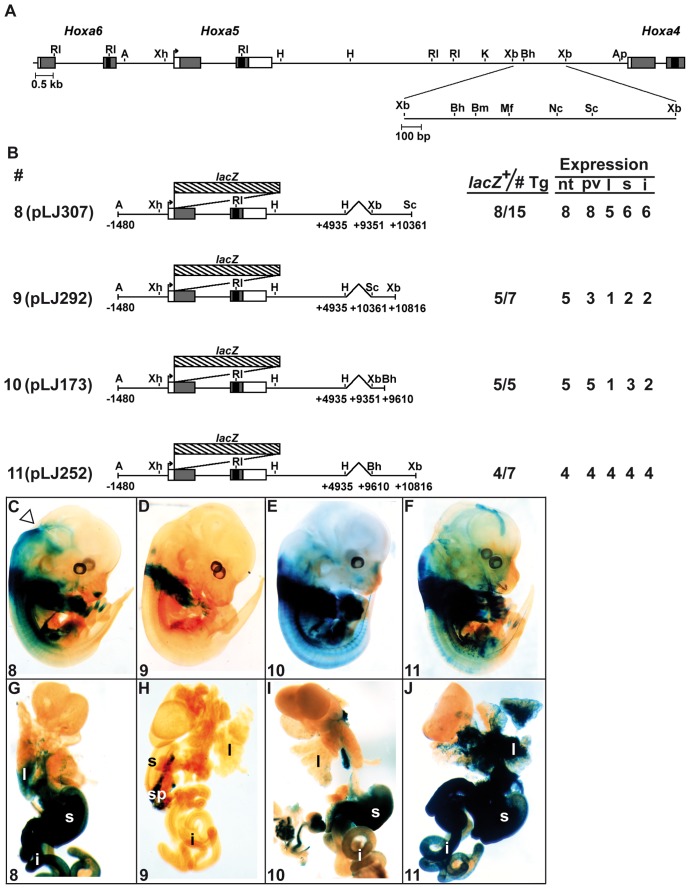
Characterization of the 1.5-kb *Xba*I-*Xba*I DNA fragment in E13.5 F0 transgenic mouse embryos. (A) Schematic representation of the 1.5-kb *Xba*I-*Xba*I DNA fragment in the *Hoxa4-Hoxa6* genomic region. (B) Diagram of the *Hoxa5/lacZ* constructs used to generate E13.5 F0 transgenic embryos and summary of transgenic expression analyses. (C–F) Carcass of representative E13.5 transgenic embryos and the associated organs (G–J) stained for β-galactosidase activity showed the effects of the different deletions on the expression pattern. Open arrowhead points the anterior limit of transgene expression in the neural tube. i, intestine; l, lung; nt, neural tube; pv, prevertebrae; s, stomach; sp, spleen.

To narrow down the organ-specific regulatory sequences, a deletion analysis was undertaken (Fig. 3). A 1.0-kb *Xba*I-*Sac*I fragment (construct 8) targeted organ and CNS expression similarly to construct 4 suggesting that the 455-bp located at the 3′ end of the *Xba*I-*Xba*I sequence were not necessary (Fig. 3C, G). This was confirmed with construct 9 containing the 455-bp *Sac*I-*Xba*I sequence. The expression profile of construct 9 was identical to that of construct 2 demonstrating the lack of regulatory activity in the 455-bp sequence (Fig. 3D, H). Additional deletion of sequences at the 3′ end of the *Xba*I-*Xba*I sequence showed that the 259-bp *Xba*I-*Bss*HII fragment (construct 10) directed expression in the stomach and intestine in few specimens but did not reproduce the anterior boundary in the hindbrain and the lung expression, suggesting the loss of tissue-specific elements (Fig. 3E, I). The reverse construct carrying the 1.2-kb *Bss*HII-*Xba*I sequence (construct 11) targeted organ expression as construct 4. However, expression in CNS was not entirely recovered suggesting that neural-specific sequences were spread along the 1.0-kb *Xba*I-*Sac*I region (Fig. 3F, J).

**Figure 4:**
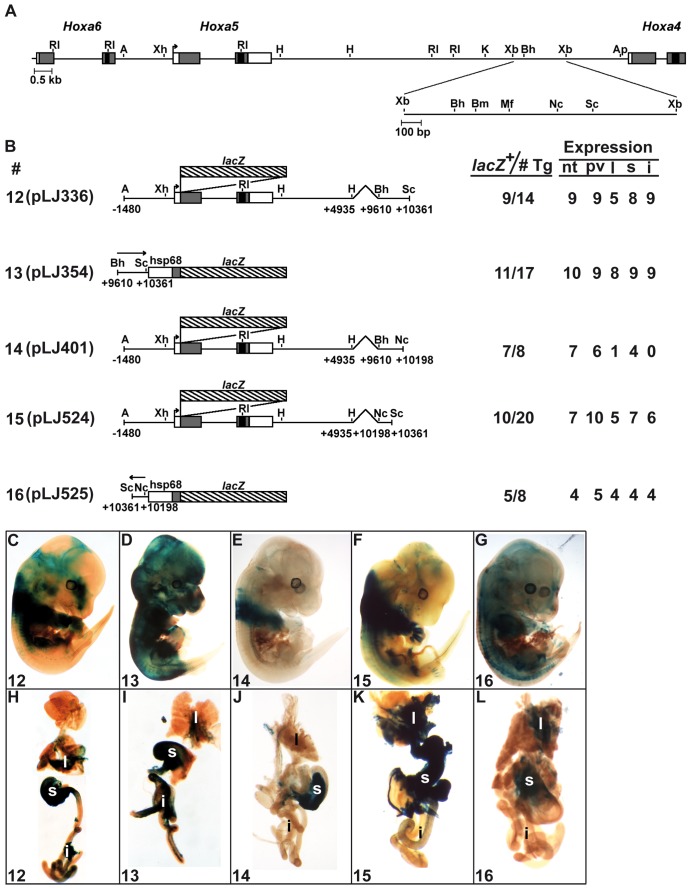
Characterization of the 751-bp *Bss*HII-*Sac*I DNA fragment in E13.5 F0 transgenic mouse embryos. (A) Schematic representation of the 1.5-kb *Xba*I-*Xba*I DNA fragment in the *Hoxa4-Hoxa6* genomic region. (B) Diagram of the *Hoxa5/lacZ* constructs used to generate E13.5 F0 transgenic embryos and summary of transgenic expression analyses. (C–G) Carcass of representative E13.5 transgenic embryos and the associated organs (H–L) stained for β-galactosidase activity showed the effects of the different deletions on the expression pattern. i, intestine; l, lung; nt, neural tube; pv, prevertebrae; s, stomach.

X-Gal staining in organs was similar in transgenic embryos carrying constructs 4, 8 and 11, suggesting that organ tissue-specific sequences were included into the 751-bp *Bss*HII-*Sac*I region. This was confirmed with construct 12 that showed expression in the respiratory and digestive tracts (Fig. 4B–C, H). Interestingly, when linked to the heterologous *hsp68*/*lacZ* plasmid, the 751-bp *Bss*HII-*Sac*I region targeted expression in organs in contrast to what was seen with constructs 6 and 7, suggesting the presence of repressive sequences in the 259-bp *Xba*I-*Bss*HII and/or in the 455-bp *Sac*I-*Xba*I DNA fragments (construct 13; Fig. 4D, I). Thus, the 751-bp *Bss*HII-*Sac*I fragment contains enhancer sequences that direct organ-specific expression.

Further deletions were performed. The 588-bp *Bss*HII-*Nco*I sequence in construct 14 did not correctly reproduce the staining in organs pinpointing toward the 163-bp *Nco*I-*Sac*I sequence as the one carrying the organ-specific enhancer (Fig. 4E, J). This was confirmed with the *Nco*I-*Sac*I sequence in construct 15, which showed organ expression in transgenic embryos (Fig. 4F, K). A similar result was observed when the *Nco*I-*Sac*I sequence was tested with the heterologous *hsp68*/*lacZ* plasmid (construct 16; Fig. 4G, L). Thus, organ-specific regulatory elements appear to be dispersed along the 1.5-kb *Xba*I-*Xba*I region, some located in the 259-bp *Xba*I-*Bss*HII fragment, others in the 163-bp *Nco*I-*Sac*I sequence.

### The RARE sequence is not necessary for *Hoxa5* organ-specific expression

**Figure 5:**
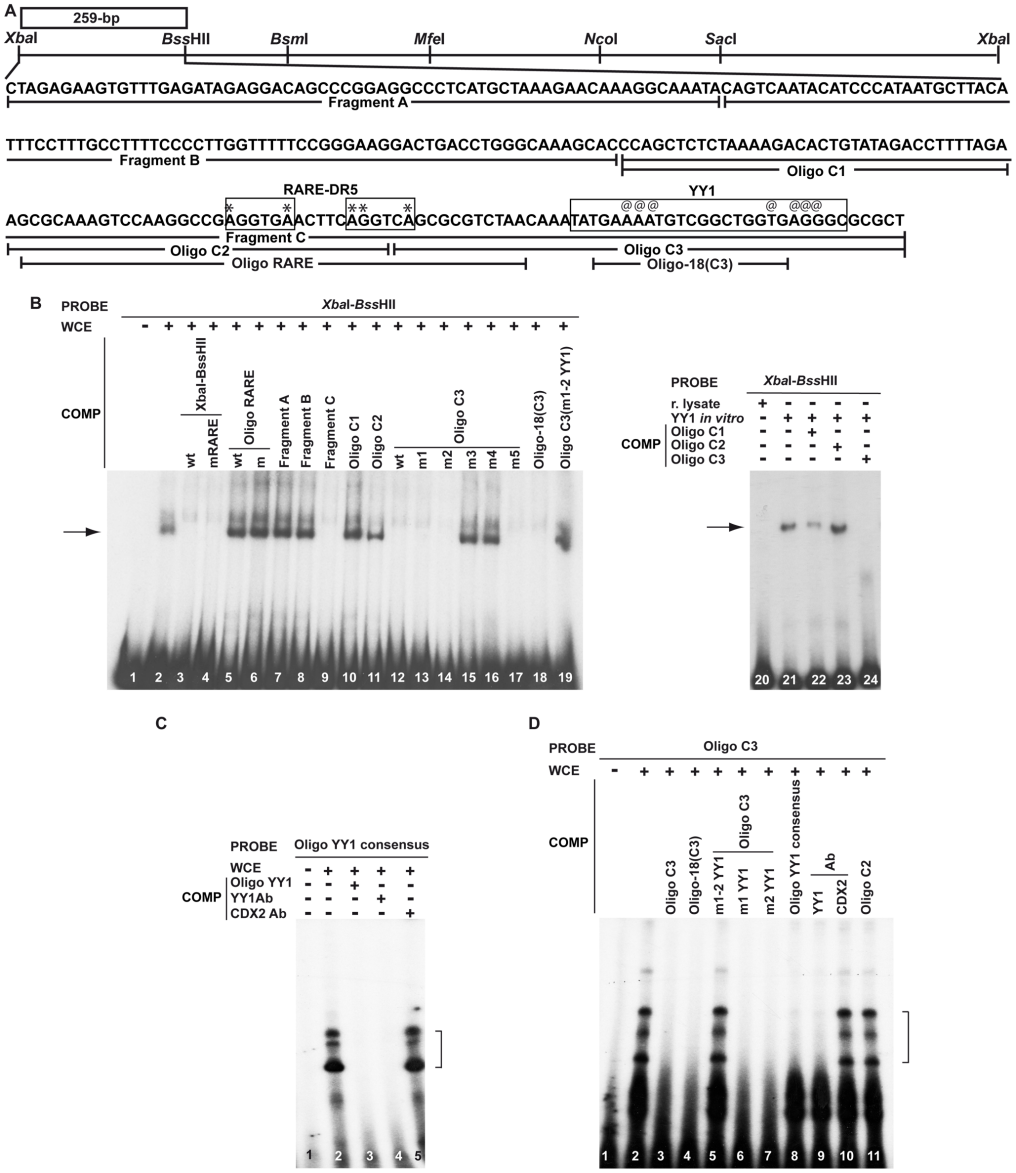
Characterization of the 259-bp *Xba*I-*Bss*HII DNA fragment by EMSA. (A) Restriction map of the 1.5-kb *Xba*I-*Xba*I sequence extending from +9.3-kb to +10.8-kb in the 3′ half of the *Hoxa4*-*Hoxa5* intergenic region. The box denotes the location of the 259-bp *Xba*I-*Bss*HII regulatory region. Sequence of the 259-bp *Xba*I*-Bss*HII DNA fragment is indicated. Fragments A, B and C used as competitor in EMSA are underlined. Fragment C was further subdivided into Oligos C1, C2, C3 and Oligo RARE and Oligo-18(C3). Boxed nucleotides correspond to RARE-DR5 sequence and YY1 binding sites. Symbols * and @ indicate point mutations into RARE and YY1 binding sites, respectively (see Table 1B for nucleotide sequences). (B) EMSA with WCE from lung/stomach/intestine of E13.5 embryos and the 259-bp *Xba*I-*Bss*HII radiolabelled probe in presence of unlabelled competitors in 100-fold excess showed that protein binding occurred with the Oligo-18(C3) fragment via YY1 binding sites (lanes 9, 12, 18–19). No binding with the RARE site was observed (lanes 4-6). EMSA with *in vitro*-translated YY1 protein and the 259-bp *Xba*I-*Bss*HII probe showed specific binding that was competed by Oligo C3 (lanes 21–24). (C) The binding of WCE with YY1 consensus binding site and the loss of binding when the YY1 antibody was added confirmed the presence of YY1 protein in WCE (lanes 1–5). (D) EMSA with WCE and Oligo C3 radiolabelled probe showed binding that was competed by an excess of cold Oligo C3, Oligo-18(C3) sequence, the YY1 consensus sequence, localized mutations in YY1 sites, and the addition of the YY1-specific antibody (lanes 1–4, 6–9). No competition occurred when a non-specific probe was used (Oligo C2), when several mutations were distributed along the YY1 binding sites in Oligo C3 or when the CDX2 control antibody was used (lanes 5, 10–11). Arrows and brackets indicate the bands corresponding to YY1 binding. COMP, competitor; r. lysate, reticulocyte lysate.

To gain insight into the critical DNA sequences involved in organ-specific expression, we investigated whether the 259-bp *Xba*I-*Bss*HII and 163-bp *Nco*I-*Sac*I DNA regions contain binding sites for putative transcriptional regulators. The *Xba*I-*Bss*HII fragment includes a previously identified RARE-DR5 required for *Hoxa4* RA-responsiveness in CNS and for *Hoxa4* lung and stomach expression (Fig. 5A) [44]. Genomic sequence comparison with the zebrafish *HoxAa* cluster also revealed that this RARE was part of a DNA region of high homology (>70%) spanning ∼100-bp, suggesting the presence of evolutionary conserved regulatory elements (not shown) [45]. To define if the RARE sequence was involved in *Hoxa5* regulation during organogenesis, we performed EMSA with WCE from lung, stomach and intestine of E13.5 mouse embryos and a 259-bp *Xba*I-*Bss*HII radiolabelled probe. Binding was observed and the specificity was confirmed by competition with a 100-fold excess of unlabelled probe. However, competition was also detected when the RARE site was mutated (Fig. 5B, lanes 1–4). Moreover, no competition occurred with oligos containing wt or a mutated version of the RARE (Fig. 5B, lanes 5–6).

**Figure 6:**
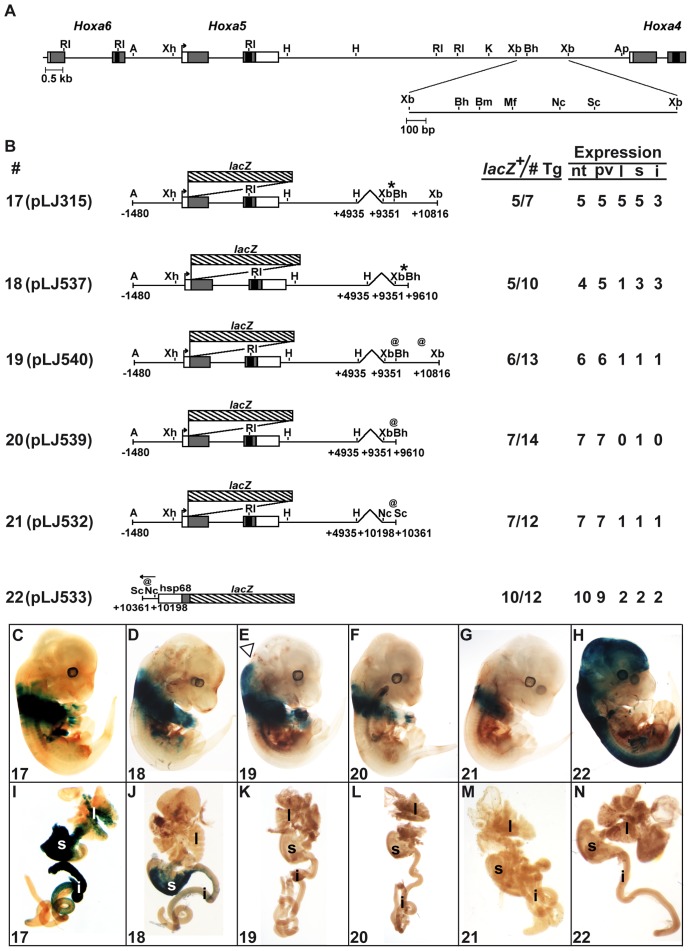
Characterization of the RARE and YY1 binding sites in E13.5 F0 transgenic mouse embryos. (A) Schematic representation of the 1.5-kb *Xba*I-*Xba*I DNA fragment in the *Hoxa4-Hoxa6* genomic region. (B) Diagram of the *Hoxa5/lacZ* constructs used to generate E13.5 F0 transgenic embryos and summary of transgenic expression analyses. The asterisks in constructs 17–18 and the @ symbols in constructs 19–22 correspond to mutations in the RARE and YY1 binding sites, respectively. (C–H) Carcass of representative E13.5 transgenic embryos and the associated organs (I–N) stained for β-galactosidase activity showed the effects of the mutations on the expression pattern. Open arrowhead points the anterior limit of transgene expression in the neural tube. i, intestine; l, lung; nt, neural tube; pv, prevertebrae; s, stomach.

We assessed the RARE regulatory activity in E13.5 *Hoxa5*/*lacZ* transgenic embryos by mutating the RARE sequence in the 1.5-kb *Xba*I-*Xba*I DNA fragment (construct 17) and in the 259-bp *Xba*I-*Bss*HII fragment (construct 18; Fig. 6B). Except for the CNS anterior boundary that was not reproduced, construct 17 targeted transgene expression in lung, stomach and intestine similarly as construct 4 (Fig. 6C, I). Likewise, construct 18 presented a comparable staining pattern than construct 10 with expression in the stomach and intestine but not in lung (Fig. 6D, J). Thus, the RARE sequence, found to be necessary to drive *Hoxa4* expression in embryonic lung and gut, does not play a key role in *Hoxa5* organ expression at E13.5.

### The YY1 transcription factor binds to *Hoxa5* organ-specific regulatory DNA regions

To identify which DNA region from the *Xba*I-*Bss*HII radiolabelled probe bound proteins in WCE from organs of E13.5 embryos, the 259-bp sequence was divided into three fragments (A to C) used as competitors. Only fragment C competed binding with the *Xba*I-*Bss*HII probe (Fig. 5B, lanes 7–9). Moreover, only the 45-bp Oligo C3 located at the 3′ end of fragment C could compete binding with the *Xba*I-*Bss*HII probe (Fig. 5B, lanes 10–12). Using a linker scanning approach, we found that an 18-bp sequence in Oligo C3 (Oligo-18(C3)) was responsible for the binding (Fig. 5B, lanes 13–18). Sequence comparison with the TFSEARCH and TESS databases revealed YY1 binding sites in Oligo-18(C3). Mutations of the YY1 sites in Oligo C3 impaired its capacity to compete with the *Xba*I-*Bss*HII probe (Fig. 5B, lane 19). To further establish that YY1 can bind the *Xba*I-*Bss*HII fragment, we used a murine YY1 protein produced *in vitro*. Binding specificity to the *Xba*I-*Bss*HII probe was confirmed and competition with Oligo C3 caused YY1 protein binding inhibition (Fig. 5B, lanes 20–24).

We then verified that our WCE contained YY1 protein by performing supershift assays. The specificity of the YY1 antibody was validated by testing an YY1 consensus sequence with WCE [28]. Addition of the YY1 antibody specifically resulted in the loss of DNA-protein complexes (Fig. 5C). A CDX2 antibody was used as negative control. In parallel, we tested the Oligo C3 sequence with WCE in EMSA. Binding was observed and it was competed by an excess of cold Oligo C3, Oligo-18(C3) and the YY1 consensus sequence (Fig. 5D, lanes 1–4, 8). No competition was observed when a non-specific probe was used (Oligo C2) or when several mutations distributed along the YY1 binding sites were inserted into Oligo C3 (Fig. 5D, lanes 5, 11). Presence of localized mutations in YY1 sites allowed competition to happen (Fig. 5D, lanes 6–7). Supershift assays were also performed with the YY1-specific antibody and the CDX2 control antibody. Only the addition of the YY1 antibody led to the loss of Oligo C3-protein complexes (Fig. 5D, lanes 9–10). Thus, the YY1 protein present in WCE from organs of E13.5 mouse embryos specifically binds a site located at the 3′ end of the 259-bp *Xba*I-*Bss*HII fragment.

**Figure 7:**
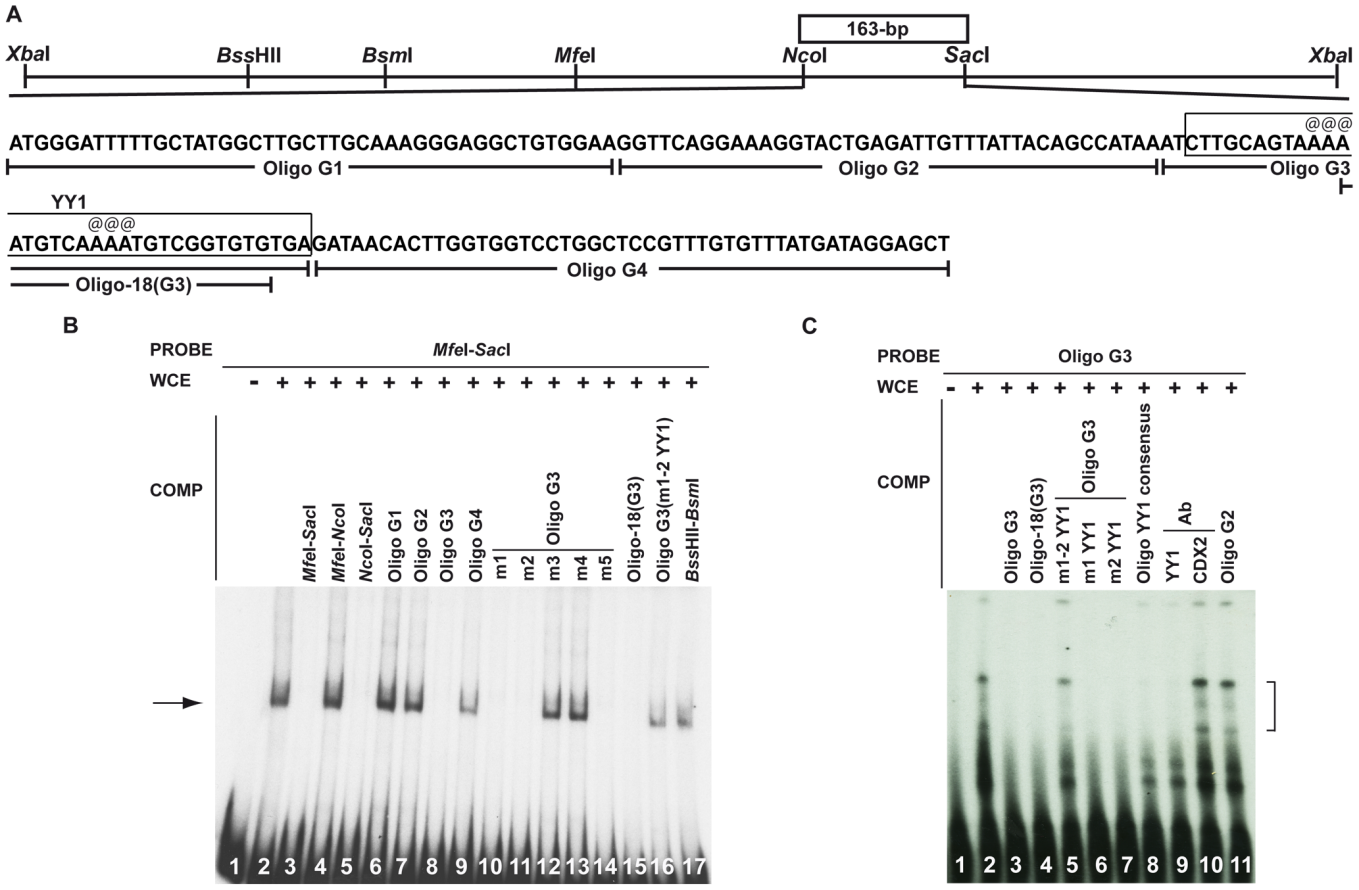
Characterization of the 163-bp *Nco*I-*Sac*I DNA fragment by EMSA. (A) Restriction map of the 1.5-kb *Xba*I-*Xba*I sequence extending from +9.3-kb to +10.8-kb in the 3′ half of the *Hoxa4*-*Hoxa5* intergenic region. The box denotes the location of the 163-bp *Nco*I-*Sac*I regulatory region. Sequence of the 163-bp *Nco*I-*Sac*I DNA fragment is indicated. Oligos G1, G2, G3, G4 and Oligo-18(G3) are underlined. Boxed nucleotides correspond to YY1 binding sites. Symbols @ indicate point mutations into YY1 binding sites (see Table 1C for nucleotide sequences). (B) EMSA with WCE from lung/stomach/intestine of E13.5 embryos and the 433-bp *Mfe*I-*Sac*I radiolabelled probe in presence of unlabelled competitors in 100-fold excess showed that protein binding occurred with the Oligo-18(G3) fragment via YY1 binding sites (lanes 8, 15–16). (C) EMSA with WCE and Oligo G3 probe showed binding that was competed by an excess of cold Oligo G3, Oligo-18(G3) sequence, the YY1 consensus sequence, localized mutations in YY1 sites, and the addition of the YY1-specific antibody (lanes 1–4, 6–9). No competition occurred when a non-specific probe was used (Oligo G2), when several mutations were distributed along the YY1 binding sites in Oligo G3 or when the CDX2 control antibody was used (lanes 5, 10–11). Arrows and brackets indicate the bands corresponding to YY1 binding. COMP, competitor.

A similar systematic approach was applied to decipher the regulatory elements of the 163-bp *Nco*I-*Sac*I sequence. EMSAs combining the 433-bp *Mfe*I-*Sac*I radiolabelled probe with WCE demonstrated specific binding with the 163-bp *Nco*I-*Sac*I portion (Fig. 7B, lanes 1–5). The latter was further divided into four oligos, leading to the identification of the 45-bp Oligo G3 as the binding sequence. Using the linker scanning approach, an 18-bp sequence in oligo G3 (Oligo-18(G3)) was found to be necessary for protein binding (Fig. 7B, lanes 6–15). Sequence comparisons with databases revealed YY1 binding sites in Oligo-18(G3), which when mutated in Oligo G3 impaired its capacity to compete protein binding with the 433-bp *Mfe*I-*Sac*I sequence as the non-specific *Bss*HII-*Bsm*I competitor (Fig. 7B, lanes 16–17). We also tested the Oligo G3 sequence with WCE, the YY1-specific antibody and various competitors and we confirmed that the YY1 protein present in WCE from organs of E13.5 mouse embryos can specifically bind the 163-bp *Nco*I-*Sac*I sequence (Fig. 7C; lanes 1–11).

### Functional YY1 binding sites are involved in *Hoxa5* organ-specific expression

**Figure 8:**
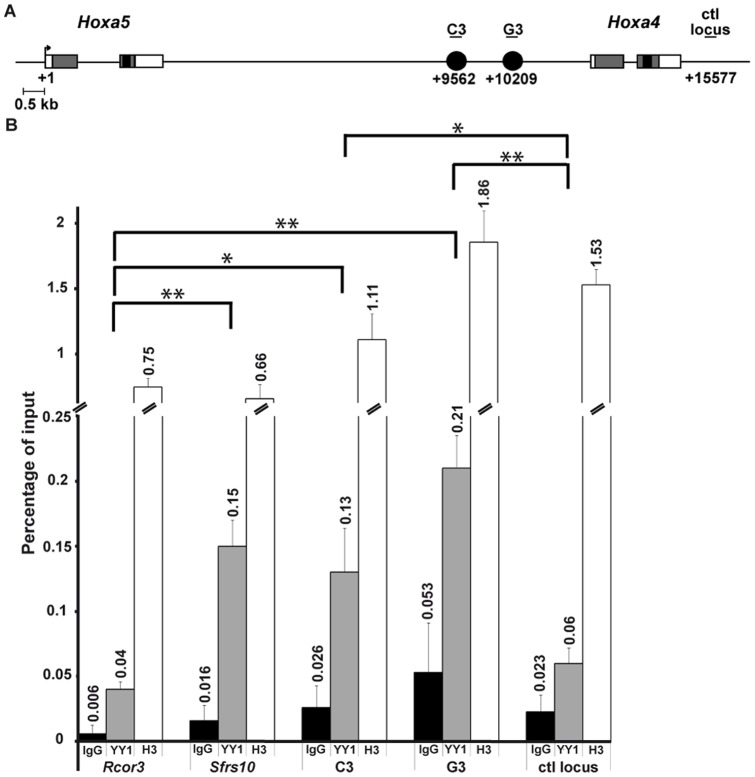
*In vivo* detection of YY1 protein binding to Oligo C3 and Oligo G3 regions by ChIP analysis. (A) Schematic representation and position relative to the *Hoxa5* TSS of the two YY1 binding sites in the *Hoxa4-Hoxa5* intergenic region. The black circles represent the Oligo C3 and Oligo G3 sequences identified by EMSA. The position of the qPCR fragments corresponding to Oligo C3, Oligo G3 and a *Hox* control locus located 15-kb downstream the *Hoxa5* TSS that does not contain YY1 binding sites (ctl locus) is indicated. (B) ChIP analysis of the *Hoxa4-Hoxa5* intergenic region in lungs from E13.5 mouse embryos. Chromatin was immunoprecipitated with rabbit IgG (negative control), anti-YY1 and anti-histone H3 (for chromatin integrity control) antibodies. Recruitment of YY1 and histone H3 on Oligo C3 and Oligo G3 sequences, an YY1 negative control (*Rcor3*), an YY1 positive control (*Sfrs10*), and the *Hox* control locus was evaluated by qPCR and is indicated as the percentage of input. The data are mean ± SEM of three independent experiments. *p<0.05, **p<0.01.

To establish whether the YY1 binding sites identified in the 259-bp *Xba*I-*Bss*HII and 163-bp *Nco*I-*Sac*I DNA regions are effective *in vivo*, a ChIP assay was performed on cross-linked chromatin isolated from either lung or stomach of E13.5 wt mouse embryos. DNA from the immunoprecipitate was subjected to qPCR analyses with specific primers for the 259-bp *Xba*I-*Bss*HII and 163-bp *Nco*I-*Sac*I sequences, for a reported YY1 target gene used as positive control (*Srfs10*), and a known YY1 negative control (*Rcor3*) [46]. A downstream region located at 15-kb of *Hoxa5* TSS and devoid of YY1 binding sites was used as a negative control for the locus. In lung and stomach, YY1 was recruited to *Hoxa5* C3 and G3 regulatory sequences and to the positive *Srfs10* control whereas no binding was observed with the *Rcor3* and *Hoxa5* locus negative controls (Fig. 8; not shown). Thus, YY1 can bind to *Hoxa5* organ-specific regulatory DNA regions both *in vitro* and *in vivo*.

We then assessed the contribution of the YY1 binding sites to *Hoxa5* organ expression in E13.5 transgenic embryos. Construct 19, which contained mutations in the YY1 binding sites of the *Xba*I-*Bss*HII and the *Nco*I-*Sac*I regions, correctly directed expression in the CNS as construct 4, while no organ expression was detected (Fig. 6E, K). Similarly, mutations of the YY1 binding sites in the *Xba*I-*Bss*HII sequence in construct 20 and in the *Nco*I-*Sac*I sequence in construct 21 caused a loss of organ expression when compared to constructs 10 and 15, respectively (Fig. 6F–G, L–M). A comparable result was observed when the *Nco*I-*Sac*I sequence with mutated YY1 sites was tested in front of the heterologous *hsp68*/*lacZ* plasmid (construct 22; Fig. 6H, N). In summary, YY1 acts as a positive regulator of *Hoxa5* lung, stomach and intestine expression during embryogenesis.

### Inactivation of *Yy1* function in mesenchyme impacts on lung formation and on *Hox* gene expression

In mice, the complete loss of *Yy1* gene function resulted in peri-implantation lethality, while the phenotypes associated with the *Yy1* conditional and hypomorphic alleles revealed a critical gene dosage requirement for YY1 during embryogenesis [38], [47]. Indeed, *Yy1* ^Floxneo/-^ mice express 25% of normal YY1 protein levels and a high percentage of newborn pups die at birth from lung defects reminiscent to those observed in *Hoxa5* ^-/-^ mice [6], [38]. To assess *in vivo* the requirement of *Yy1* gene function in *Hoxa5* lung expression, we generated *Yy1* ^Floxneo/-^ mouse embryos carrying the *Hoxa5/lacZ* construct 1 transgene. No change in *lacZ* expression pattern and *Hoxa5* RNA levels was observed in the lungs from E13.5 and E18.5 *Yy1* ^Floxneo/-^ *Tg^Hoxa5/lacZ^* ^#1^ embryos (not shown). The lack of effect on *Hoxa5* expression might result from the remaining 25% of *Yy1* expression.

**Figure 9:**
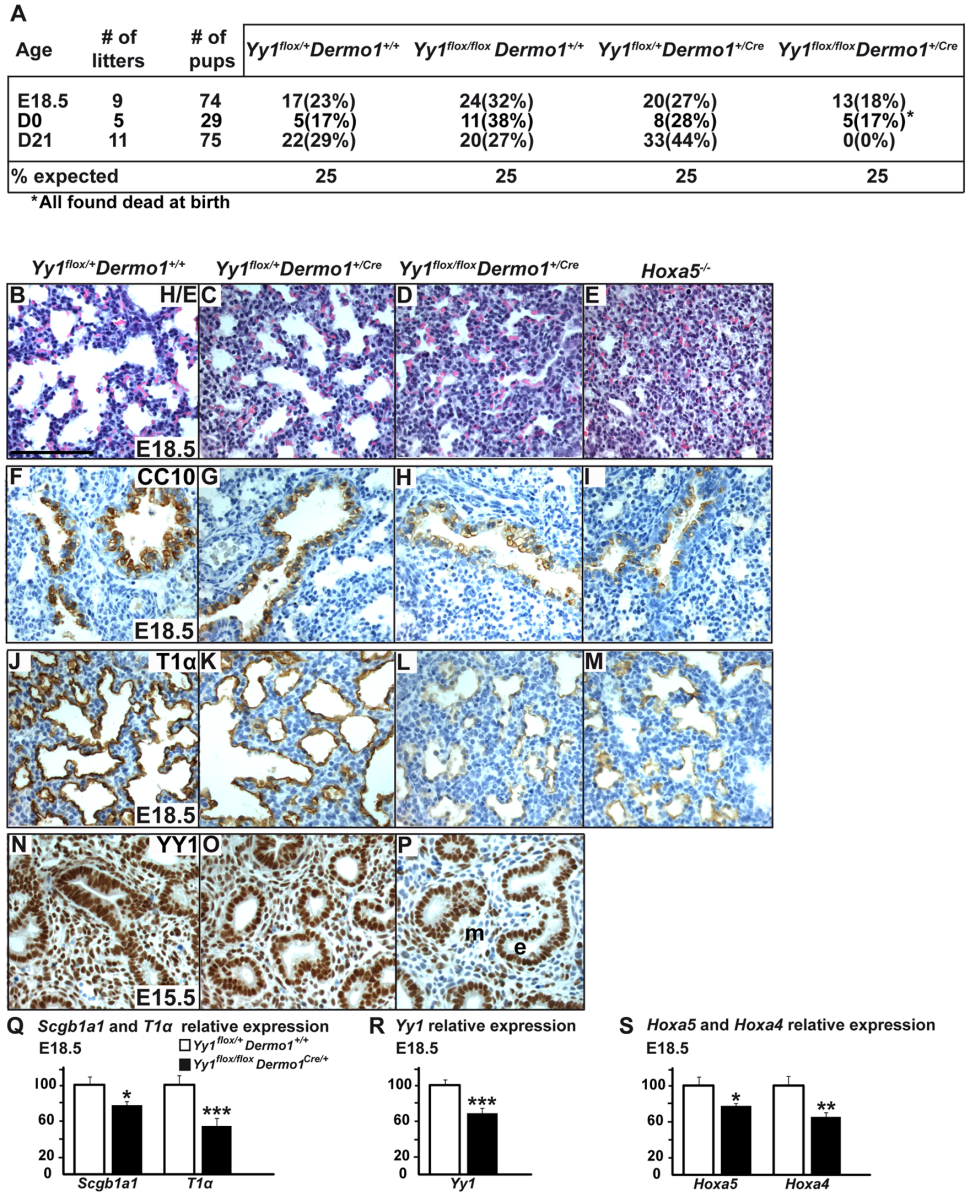
Analysis of the lung phenotype in *Yy1* ^flox/flox^ *Dermo1* ^+/Cre^ mutants. (A)Ratios of genotypes of litters obtained from matings between *Yy1* ^flox/+^ *Dermo1* ^+/Cre^ and *Yy1* ^flox/flox^ mice. (B–E) Comparative lung histology of E18.5 *Yy1* ^flox/+^ *Dermo1* ^+/+^, *Yy1* ^flox/+^ *Dermo1* ^+/Cre^, *Yy1* ^flox/flox^ *Dermo1* ^+/Cre^ and *Hoxa5* ^-/-^ embryos. *Yy1* ^flox/+^ *Dermo1* ^+/+^ and *Yy1* ^flox/+^ *Dermo1* ^+/Cre^ specimens presented a normal lung structure, whereas lungs from *Yy1* ^flox/flox^ *Dermo1* ^+/Cre^ and *Hoxa5* ^-/-^ embryos were collapsed. (F–M) Characterization of the respiratory epithelium of E18.5 *Yy1* ^flox/flox^ *Dermo1* ^+/Cre^ embryos. (F–I) Detection of club cells by CC10 immunostaining showed decreased labelling in lungs from *Yy1* ^flox/flox^ *Dermo1* ^+/Cre^ and *Hoxa5* ^-/-^ specimens. (J–M) Immunostaining with T1α, a marker of type I pneumocytes, was reduced in lungs from *Yy1* ^flox/flox^ *Dermo1* ^+/Cre^ and *Hoxa5* ^-/-^ embryos. (N–P) YY1 immunostaining showed ubiquitous YY1 expression in lung epithelial and mesenchymal compartments in E15.5 control embryos, but an important decreased staining in lung mesenchyme from *Yy1* ^flox/flox^ *Dermo1* ^+/Cre^ specimens. e, epithelium; m, mesenchyme. Scale bar: 200 μm. (Q–S) qRT-PCR analysis for *Scgb1a1*, *T1α*, *Yy1*, *Hoxa5* and *Hoxa4* expression in lungs from E18.5 *Yy1* ^flox/+^ *Dermo1* ^+/+^ and *Yy1* ^flox/flox^ *Dermo1* ^+/Cre^ embryos. Expression levels were significantly diminished for all genes tested in *Yy1* ^flox/flox^ *Dermo1* ^+/Cre^ specimens. Values are expressed as means ± SEM. *p<0.05, **p<0.01, ***p<0.001.

As *Hoxa5* is specifically expressed in lung mesenchyme, we inactivated *Yy1* gene function in the mesenchyme using the *Dermo1* ^Cre^ deleter mouse line [35], [39]. No *Yy1* ^flox/flox^ *Dermo1* ^+/Cre^ animals were recovered at weaning and monitoring of litters showed that all *Yy1* ^flox/flox^ *Dermo1* ^+/Cre^ pups were found dead at birth (Fig. 9A). At E18.5, the distribution of genotypes was conformed to the expected mendelian ratio. Thus, mesenchymal *Yy1* deletion causes death at birth.

Comparative lung morphology of E18.5 embryos revealed that *Yy1* ^flox/+^ *Dermo1* ^+/+^ and *Yy1* ^flox/+^ *Dermo1* ^+/Cre^ specimens presented a normal structure with dilated peripheral lung saccules and thin mesenchyme. However, lungs from *Yy1* ^flox/flox^ *Dermo1* ^+/Cre^ embryos showed a collapsed appearance with narrow airspaces and thick mesenchyme similar to the *Hoxa5^-/-^* lung phenotype, a likely explanation for the neonatal lethality of *Yy1* mutant pups (Fig. 9B–E). We also assessed the impact of the mesenchymal *Yy1* deletion on the integrity of the airway epithelium by looking at the expression of cell specific markers by qRT-PCR and IHC. A statistically decreased expression of the club cell (Clara cell) marker CC10 (encoded by the *Scgb1a1* gene) was observed in lung specimens from E18.5 *Yy1* ^flox/flox^ *Dermo1* ^+/Cre^ embryos by qRT-PCR (Fig. 9Q). IHC analysis showed that the decreased CC10 staining paralleled that seen in *Hoxa5* ^-/-^ specimens (Fig. 9F–I) [41]. As well, expression of podoplanin (T1α), a marker of type I pneumocytes, which are participating to gas exchanges with the underlying vascular endothelial cells, was decreased in *Yy1* mutant specimens similar to what was previously observed in *Hoxa5* ^-/-^ mutants (Fig. 9J–M, Q) [35]. Together, these results indicated that the *Yy1* mesenchymal deletion perturbed epithelial cell differentiation along the respiratory tract, a phenotype reminiscent to that of *Hoxa5* mutants.

We validated the specific inactivation of *Yy1* in lung mesenchyme by IHC analysis. At E15.5 and E18.5, the YY1 protein showed ubiquitous expression in lung epithelial and mesenchymal compartments from control embryos, whereas in *Yy1* ^flox/flox^ *Dermo1* ^+/Cre^ specimens, YY1 staining was greatly diminished in lung mesenchyme but unchanged in the epithelium (Fig. 9O–P; not shown). qPCR analysis confirmed the decreased *Yy1* expression in lungs from E18.5 *Yy1* ^flox/flox^ *Dermo1* ^+/Cre^ embryos (Fig. 9R). We also looked at the impact of the *Yy1* mesenchymal deletion on *Hoxa5* and *Hoxa4* lung expression as the 1.5-kb *Xba*I-*Xba*I DNA fragment containing the YY1 binding sites was shown to be necessary for *Hoxa4* lung and gut expression [44]. qRT-PCR expression analysis revealed significantly reduced *Hoxa5* and *Hoxa4* expression levels in lungs from E18.5 *Yy1* ^flox/flox^ *Dermo1* ^+/Cre^ embryos, indicating that an integrated regulation of *Hoxa4* and *Hoxa5* genes prevailed in lung expression through the sharing of YY1-responsive sequences.

## Discussion

Our search for *Hoxa5* transcriptional regulatory sequences led us to identify a 14.5-kb genomic fragment (construct 1) encompassing the *Hoxa5* gene and starting into the homeobox of the 5′ flanking *Hoxa6* gene up to ∼350-bp upstream of the *Hoxa4* TSS. The 14.5-kb transgene reproduces the spatio-temporal expression directed by the *Hoxa5* proximal promoter by targeting *lacZ* expression to the brachial region of the CNS and to the paraxial and lateral plate mesoderm at the cervico-thoracic level as reported for the shorter version of 11.1-kb in length [31]. In addition, the 14.5-kb transgene recapitulates *Hoxa5* expression in the mesenchymal compartment of the respiratory and digestive tracts, the *Hoxa5* rostro-caudal gradient in the stomach, and the anterior limit of *Hoxa5* expression in the CNS at the level of the floor of myencephalon [6]–[8], [31].

*Hoxa5* expression in the developing neural tube is under the control of the BSC enhancer, which directs expression in the brachial region of the spinal cord from E11 to E13, a subset of the *Hoxa5* endogenous pattern in the neural tube [30]. We now report that sequences distributed in the 1.5-kb *Xba*I-*Xba*I DNA fragment replicate the anterior expression domain of *Hoxa5* in the neural tube. However, the onset of *Hoxa5* expression in the developing CNS up to E11 is not reproduced with the 14.5-kb transgene indicating that additional sequences are required [31]. Moreover, mutation of the RARE site located in the 1.5-kb *Xba*I-*Xba*I fragment causes a caudal shift of the anterior boundary in the CNS suggesting that RA signaling is involved in the establishment of the correct *Hoxa5* expression domain in the neural tube as shown for other *Hox* genes [48]–[49]. Interestingly, when tested in a *Hoxa4* context, mutation of the RARE sequence did not impact on the activation of *Hoxa4* embryonic expression in the developing neural tube but it affects *Hoxa4* response to exogenous RA in the neural tube [44]. Thus, the *Hoxa4* and *Hoxa5* genes share a RARE site that positively regulates their respective expression in the developing CNS. This RARE sequence is highly conserved between *Hox* clusters and between vertebrates. It was shown to possess neural enhancer activity for the *Hoxd4* gene [49]–[51]. In the *HoxB* cluster, the *Hoxb5* distal RARE was found to regulate the anterior expression boundary of 5′ *Hoxb* genes in the posterior hindbrain raising the possibility that the RARE site located in the 1.5-kb *Xba*I-*Xba*I fragment may play a similar evolutionarily conserved role in the *HoxA* complex [23].

While the RARE site in the 1.5-kb *Xba*I-*Xba*I fragment appears important for *Hoxa5* expression in CNS, it is not required for *Hoxa5* expression in the developing lung and gut when tested in E13.5 transgenic embryos. In EMSA experiments with the 259-bp *Xba*I-*Bss*HII fragment, the lack of binding between the RARE sequence and WCE prepared from lung, stomach and intestine of E13.5 mouse embryos further supports the absence of a role for the RARE sequence in *Hoxa5* organ expression. In contrast, *Hoxa4* expression in lung, gut and metanephros at the same embryonic age is dependent on the functional RARE, suggesting that the action of the RARE site is *Hox* promoter-specific [44]. We cannot rule out the possibility that the RARE site may be functional in a time-dependent manner regulating *Hoxa5* lung and gut expression at earlier embryonic stages as suggested by studies showing that RA deficiency negatively impacts on *Hoxa5* expression in the developing lung and stomach at E10.5 [52]. Another RARE located at the 3′ end of the human *HOXA5* gene was shown to mediate RA responsiveness of the gene in breast cancer cells [53]. This RARE site is conserved in the mouse genome at position +7.4-kb from the *Hoxa5* TSS. Even though our deletion studies did not identify the corresponding genomic region to be involved in *Hoxa5* gene regulation, we cannot exclude a role for this RARE in *Hoxa5* developmental expression.

Our combined approach of transgenesis and biochemistry revealed that YY1 is a positive regulator of *Hoxa5* gene expression in the developing respiratory and digestive tracts, while it does not play a major role in CNS expression. YY1 is a multifunctional zinc-finger-containing transcription factor, identified as the homolog of *Drosophila* Pleiohomeotic (PHO) protein, the latter recruiting Polycomb group (PcG) proteins to negatively regulate genes. YY1 plays crucial roles in numerous biological processes by selectively initiating, activating or repressing transcription, depending upon promoter contextual differences or specific protein interactions [54]. The role of YY1 in *Hox* gene regulation is mainly associated with repression as shown for the *Hoxb4*, *Hoxd4*, *HOXB13* and *HOXD11-HOXD12* genes. For instance, YY1 binds *Hoxb4* promoter and intron enhancer sequences through overlapping NFY/YY1 sites suggesting that the relative levels of binding of the transcriptional activator NFY and YY1 through the same site mediate opposing transcriptional effects on *Hoxb4* expression along the A-P axis [26]. YY1 represses *Hoxd4* expression in undifferentiated P19 cells by recruiting the PcG protein MEL18 to the *Hoxd4* proximal promoter, which maintains silencing at the *Hoxd4* locus [28]. YY1 also participates in the repression of *HOXB13* expression in prostate cancer cells through an epigenetic mechanism involving histone acetylation modification [55]. Finally, YY1 binding sites are present in the *HOXD11*-*HOXD12* intergenic region in human embryonic stem cells, YY1 recruiting PcG proteins for the transcriptional repression of the distal *HOXD* genes [29]. So far, only the *Hoxb7* gene expression is positively regulated by YY1 in tumor and transformed cell lines [27].

Here, our work established for the first time the positive role of YY1 in *Hox* gene expression in a normal context, the embryo. Our EMSA and ChIP data obtained with organ preparations from E13.5 embryos demonstrated that YY1 physically interacts with two *Hoxa5* regulatory sequences located in the *Hoxa5*-*Hoxa4* intergenic region. Mutations in these YY1 binding sites abolished *Hoxa5* expression in lung, stomach and intestine from transgenic embryos. Moreover, the conditional deletion of *Yy1* function in lung mesenchyme resulted in decreased levels of *Hoxa5* transcripts in the developing lung. Altogether, these data demonstrated that YY1 acts as a transcriptional activator of *Hoxa5* expression in lung and gut during embryogenesis.

In the trunk of E12.5 mouse embryos, YY1 binding sites are present in *Hoxa5* upstream sequences and they co-localize with EED and BMI1 binding sites, two PcG protein members of the PcG repressive complex 1. PcG binding is specific to the anterior domain of the trunk and it results in the transcriptional silencing of *Hoxa5* in this axial region demonstrating that PcG repression is involved in the establishment of the correct *Hoxa5* expression domain in the prevertebral column [56]. Thus, *Hoxa5* developmental expression is under the control of several YY1 binding sites distributed along the *Hoxa5* locus. Depending on the developmental context, YY1 can mediate PcG repression of *Hoxa5* expression via binding sites located in *Hoxa5* upstream sequences to finely define *Hoxa5* expression domain along the A-P axis [56]. Here, we showed that YY1 can also activate *Hoxa5* expression in the developing respiratory and digestive tracts acting via binding sites located in the intergenic *Hoxa5*-*Hoxa4* region and shared with the flanking *Hoxa4* gene. Studies have shown that YY1 acts by recruiting co-activators to function on YY1-activated targets [57]–[61]. This situation may also prevail for the *Hoxa5* gene. While some of these coactivators are expressed in the lung (INO80, PRMT1, BAP1, GATA-4, AP-1), no information is available about their potential role in lung development.

Genetic analyses have revealed the crucial role played by YY1 during embryogenesis. *Yy1* ^-/-^ embryos die shortly after implantation [47]. The use of the *Yy1* conditional allele with lineage-specific *Cre* mouse lines circumvents the early embryonic lethality of *Yy1* null mutants revealing the large spectrum of YY1 actions throughout life [38], [62]–[65]. A dosage-dependent requirement for YY1 is essential for survival as newborn mice expressing 25% of normal YY1 levels die at birth from respiratory failure [38]. Despite these observations, little is known about the *Yy1* function during lung morphogenesis. Here, we showed that the specific ablation of *Yy1* function in mesenchyme via the use of *Dermo1* ^+/Cre^ deleter mice causes neonatal death of mutant pups likely due to lung defects. Lungs from *Yy1* ^flox/flox^ *Dermo1* ^+/Cre^ embryos were collapsed with narrow airspaces and thick mesenchyme. Epithelial cell differentiation along the respiratory tract was also affected as shown by the decreased expression of CC10 and T1α, specific markers of club cells present in the respiratory airways and type I pneumocytes lining the alveolar epithelium, respectively. These lung phenotypes were similar to those observed in *Hoxa5^-/-^* embryos, supporting the notion that mesenchymal YY1 action during lung formation is mediated, at least in part, by the control of *Hoxa5* expression [6], [35], [41]. This is further reinforced by the decreased *Hoxa5* expression in lungs from *Yy1* ^flox/flox^ *Dermo1* ^+/Cre^ embryos. However, according to the broad transcriptional activity of YY1, the lung phenotype of *Yy1* ^flox/flox^ *Dermo1* ^+/Cre^ mutants may also result from the deregulation of other genes.

YY1 was shown to be essential for the transcription of the *Scgb1a1* gene in endometrial cells [66]. In the present case, the decreased *Scgb1a1* expression observed in the respiratory epithelium of *Yy1* ^flox/flox^ *Dermo1* ^+/Cre^ embryos is a non-cell autonomous phenotype since the *Yy1* mutation is specific to mesenchyme. *Scgb1a1* expression is also diminished in the respiratory epithelium of *Hoxa5* ^-/-^ mutants [41]. Therefore, *Hoxa5* could be a downstream effector of YY1. Alternatively, *Yy1* and *Hoxa5* genes may act on *Scgb1a1* expression via distinct pathways.

*Hoxa4* lung expression was reduced in lungs from *Yy1* ^flox/flox^ *Dermo1* ^+/Cre^ embryos, suggesting that the YY1 binding sites present in the 1.5-kb *Xba*I-*Xba*I DNA fragment are involved in the regulation of both *Hoxa4* and *Hoxa5* genes. No lung phenotype was reported in *Hoxa4* ^-/-^ mutants, but it is possible that *Hoxa4* exerts a role during lung development masked by functional redundancy. Even though the decrease in *Hoxa4* and *Hoxa5* expression was modest in lungs from *Yy1* ^flox/flox^ *Dermo1* ^+/Cre^ embryos, the combined downregulation of *Hoxa4* and *Hoxa5* may participate to the lung phenotype observed in *Yy1* ^flox/flox^ *Dermo1* ^+/Cre^ mutants.

In summary, our search for regulatory sequences that correctly reproduce *Hoxa5* developmental expression has led to the identification of additional *cis*-acting elements present in the *Hoxa4*-*Hoxa5* intergenic region that are important for *Hoxa5* expression in CNS, lung, stomach and intestine. Several of these sequences are shared with the flanking *Hoxa4* gene supporting the model that coordinated regulatory mechanisms between *Hox* genes are essential for the precise function of each gene and the correct development of the embryo [15]. We have also unveiled the crucial role of YY1 as a transcriptional activator of *Hoxa5* lung and gut expression and the repercussions of the *Yy1* conditional deletion in lung mesenchyme. Further studies will explore the extent of the regulatory role of YY1 in *Hoxa5* developmental expression.
